# The effects of neurogranin knockdown on SERCA pump efficiency in soleus muscles of female mice fed a high fat diet

**DOI:** 10.3389/fendo.2022.957182

**Published:** 2022-08-22

**Authors:** Jessica L. Braun, Jisook Ryoo, Kyle Goodwin, Emily N. Copeland, Mia S. Geromella, Ryan W. Baranowski, Rebecca E. K. MacPherson, Val A. Fajardo

**Affiliations:** ^1^ Department of Kinesiology, Brock University, St. Catharines, ON, Canada; ^2^ Centre for Bone and Muscle Health, Brock University, St. Catharines, ON, Canada; ^3^ Centre for Neuroscience, Brock University, St. Catharines, ON, Canada; ^4^ Department of Health Sciences, Brock University, St. Catharines, ON, Canada

**Keywords:** calcineurin, calmodulin, sarcolipin, neuronatin, phospholamban, obesity

## Abstract

The sarco(endo)plasmic reticulum Ca^2+^ ATPase (SERCA) pump is responsible for the transport of Ca^2+^ from the cytosol into the sarcoplasmic reticulum at the expense of ATP, making it a regulator of both muscle relaxation and muscle-based energy expenditure. Neurogranin (Ng) is a small protein that negatively regulates calcineurin signaling. Calcineurin is Ca^2+^/calmodulin dependent phosphatase that promotes the oxidative fibre type in skeletal muscle and regulates muscle-based energy expenditure. A recent study has shown that calcineurin activation reduces SERCA Ca^2+^ transport efficiency, ultimately raising energy expenditure. Since the biomedical view of obesity states that it arises as an imbalance between energy intake and expenditure which favors the former, we questioned whether heterozygous Ng deletion (*Ng^+/-^*) would reduce SERCA efficiency and increase energy expenditure in female mice fed a high-fat diet (HFD). Young (3–4-month-old) female wild type (WT) and *Ng^+/-^* mice were fed a HFD for 12 weeks with their metabolic profile being analyzed using metabolic cages and DXA scanning, while soleus SERCA efficiency was measured using SERCA specific Ca^2+^ uptake and ATPase activity assays. *Ng^+/-^* mice showed significantly less cage ambulation compared to WT mice but this did not lead to any added weight gain nor changes in daily energy expenditure, glucose or insulin tolerance despite a similar level of food intake. Furthermore, we observed significant reductions in SERCA’s apparent coupling ratio which were associated with significant reductions in SERCA1 and phospholamban content. Thus, our results show that Ng regulates SERCA pump efficiency, and future studies should further investigate the potential cellular mechanisms.

## Introduction

Calcineurin, a Ca2+/calmodulin (CaM) dependent serine/threonine phosphatase, has previously been shown to promote the slow-oxidative phenotype in skeletal muscle (1–5). More specifically, in the presence of intracellular Ca2+ ([Ca2+]i), CaM will be activated *via* a Ca2+/CaM complex that can interact with calcineurin, displacing its autoinhibitory domain, allowing for its activation (6). Active calcineurin will dephosphorylate nuclear factor of activated T-cell (NFAT) which is then able to enter the nucleus and promote the oxidative gene program (1).

Promoting the slow-oxidative phenotype *via* calcineurin signaling has proven beneficial in promoting fatigue resistance (7), protecting muscle from dystrophic pathology (8, 9), and more recently, activating non-shivering thermogenesis (NST) (10) - a promising mechanism of combatting diet-induced obesity and type II diabetes. Skeletal muscle is one of the primary sites of NST and is mainly mediated through the sarco(endo)plasmic reticulum Ca2+ ATPase (SERCA) pump (reviewed by (11, 12)). The SERCA pumps transport Ca2+ ions into the sarcoplasmic reticulum and are best known for playing a role in regulating muscle relaxation. Based on its structure and binding capacity, SERCA has an optimal coupling ratio of 2 Ca2+ ions pumped into the sarco(endo)plasmic reticulum per 1 ATP hydrolyzed (13, 14) and lowering this coupling ratio can increase SERCA’s energy expenditure and combustion of metabolic substrates (15–17). This can be achieved through the binding of SERCA uncoupling proteins, sarcolipin (SLN), and the newly characterized neuronatin (NNAT), that both lower SERCA’s Ca2+ transport efficiency (18–20). Recent work by Rotter etal. (10) has shown that knocking out Regulator of Calcineurin 1 (RCAN1), an inhibitor of calcineurin signaling, increases energy expenditure and promotes resistance to diet-induced obesity in mice partly by increasing SLN content and promoting SERCA inefficiency. Thus, calcineurin signaling can influence muscle-based energy homeostasis through the SERCA pump and may be a viable therapeutic target for metabolic disorders such as obesity and diabetes.

Neurogranin (Ng) is a negative calcineurin regulator that we recently found to be expressed in skeletal muscle, specifically the soleus (21). Ng acts to reduce the availability of CaM to interact with calcineurin while also limiting the affinity of CaM for Ca2+, ultimately reducing the activation of calcineurin (5, 21–26). In muscle, heterozygous reduction of Ng (Ng+/-) activates calcineurin signaling and promotes the slow-oxidative myogenic program and fatigue resistance (27). Here, we questioned whether Ng+/- mice fed a high-fat diet (HFD) to induce obesity, which is a condition that necessitates SERCA mediated Ca2+ cycling, would have lower SERCA efficiency in muscle due to enhanced calcineurin signaling and the promotion of SLN expression and, in turn, a better metabolic phenotype of lower body mass, body fat, and improved insulin and glucose tolerance.

## Materials and methods

### Animals

A breeding colony of heterozygous Ng knockout mice (Ng+/-) and wild-type (WT) mice on a 129/Sv and C57BL/6J mixed background was established at Brock University using cryorecovered breeding pairs from the Jackson Laboratories (stock#008233). 3-4 month old female Ng+/- and WT mice were used in this study and housed in an environmentally controlled room (23-24°C) at Brock University’s animal facility with a standard 12:12 hour light:dark cycle and access to food and water ad libitum. Mice were singly housed in a Techniplast Digital Ventilated Cage 80 (DVC80) system equipped with GYM500 software that allows for 24/7 monitoring of cage ambulation and were fed a HFD consisting of 60% kcal from fat (D12492, Research Diets) for 12 weeks. At the conclusion of the diet, mice were euthanized *via* cervical dislocation while anesthetized using vaporized isoflurane. The soleus muscle, inguinal WAT (iWAT), and interscapular BAT were dissected and stored at -80°C until further analyses. All experimental protocols were approved by the Brock University Animal Care Committee (AUP 21-04-02).

### Dual-energy X-ray Absorptiometry (DXA) scanning

A small animal DXA scanner (OsteoSys InSIGHT, Scintica) was used to measure body composition in anesthetized mice (vapourized isoflurane, 5% in O2) prior to the start of the diet intervention and at 11 weeks into the diet. Percent lean and fat mass were calculated using total body mass and lean mass was used to normalize daily energy expenditure.

### Metabolic caging

To measure oxygen consumption (VO2), respiratory exchange ratio (RER), cage activity, food and water intake, mice were singly housed in a Promethion metabolic caging unit for 48 hours. The trial took place during week 10 of the diet intervention. Data was obtained for light, dark and combined (i.e., daily) cycles and VO2 was normalized to fat free mass measured with the DXA scanner.

### Novel object recognition test

The novel object recognition test was performed as described in detail by Hayward etal. (28). Briefly, mice were allowed to freely explore an empty arena (40x40x40 cm) for 10minthe day prior to testing in a prehabituation period. The following day, the habituation stage consisted of mice exploring the same arena for 10 min with two identical objects placed in opposing corners. After a 60 min delay period in their home cages, mice were returned to the arena for a 10 min testing period where one of the objects was replaced with a novel object. Time investigating the novel object was calculated and presented as a percentage of the total time investigating either object.

### Glucose and insulin tolerance tests

Whole body glucose tolerance and insulin sensitivity was measured using intraperitoneal (IP) glucose and insulin tolerance tests, respectively. Tests were performed 48 hours apart and mice had free access to their respective diets between testing. Animals were fasted for 6 hours prior to the IP glucose injection (2g/kg body weight) and tail vein blood samples were taken at 0, 15, 30, 45, 60, 90, and 120 minutes post-injection with the use of a hand-held glucometer (Freestyle Lite, Abbott). Similarly, the insulin tolerance test utilized tail vein blood samples at 0, 15, 30, 45, 60, and 90 minutes following the IP insulin injection (0.75U/kg). Plots of the average changes in glucose over time were made for each group and the total area under the curve (AUC) was calculated. AUC is presented in mmol/L*time and baseline values are set to Y=0.

### RNA extraction and real-time polymerase chain reaction

Given that the soleus was the primary muscle investigated in this study based on its oxidative nature (29) and expression of both SLN (30) and NNAT (31), RNA was extracted from the soleus and reverse transcribed into cDNA with changes in mRNA expression being determined by real-time quantitative polymerase chain reaction (RT-PCR), as previously described (32, 33). Briefly, two soleus muscles were pooled per genotype to ensure sufficient RNA concentration (total n=3 pooled pairs per group) and RNA was extracted by homogenizing in 1mL of TRIzol reagent (Ambion, 15596018) and isolated using a PureLink™ RNA Mini Kit (Invitrogen, 12183020). RT-PCR was performed using a 7500 Fast Real-Time PCR system (Applied Biosystems) with samples plated in duplicate. For Ng, a TaqMan Gene Expression Assay was used (ThermoFisher, 4331182) and GAPDH was used as a housekeeping gene (ThermoFisher, 4352932). Differences in Ng expression were determined using the 2-ΔΔCT method (34).

### SERCA Ca2+ uptake, SERCA ATPase, and coupling ratio

Rates of Ca2+ uptake in soleus muscle homogenates were performed as previously described (15, 35–37), but has recently been fitted onto a 96-well plate (20). Briefly, muscle homogenate was added to reaction buffer (200 mM KCl, 20 mM HEPES, 10 mM NaN3, 5 μ M TPEN, 15 mM MgCl2, pH 7.0) along with Indo-1 (4 μ M final concentration; 57180, Sigma-Aldrich). Samples were plated in duplicate, uptake was initiated with 10mM ATP and kinetically measured using a Molecular Devices M2 plate reader. The amount of Ca2+ taken up was measured over three time intervals of 300-600s, 600-900s, and 900-1200s, which corresponded to average pCa values of 6.10, 6.25, and 6.37. pCa was calculated as the negative logarithm of [Ca2+] after measuring free Ca2+ in our assays using Indo-1. All rates of Ca2+ uptake were normalized to protein content as measured with a bicinchoninic acid (BCA) assay.

Ca2+-dependent SERCA activity assays were measured over pCa values of 7.30-5.70 to obtain SERCA activity-pCa curves with an enzyme-linked spectrophotometric assay that indirectly measures ATP hydrolysis *via* NADH disappearance at 37°C and has been previously described in detail (38, 39). All rates were similarly normalized to protein content. In addition to the SERCA activity-pCa curves, SERCA activity was specifically measured over pCa values of 6.10, 6.25, and 6.37 in order to calculate the apparent coupling ratio (rates of Ca2+ uptake: rates of ATP hydrolysis) at matching pCa values. As with Ca2+ uptake assays, pCa was calculated as the negative logarithm of [Ca2+] after measuring free Ca2+ in our assays using Indo-1.

### Western blotting

To assess SERCA, ryanodine receptor (RYR), SLN, NNAT, and uncoupling protein 1 (UCP1) protein content in soleus muscles, iWAT and BAT, Western blotting was employed as previously described (20, 39). Briefly, soleus muscle samples were homogenized in 10X volume to weight with buffer containing 250 mM sucrose, 5 mM HEPES, 0.2 mM phenylmethylsulfonyl fluoride (PMSF), and 0.2% (wt/vol) NaN3. Adipose tissue samples were homogenized *via* FAST prep (FastPrep®, MP Biomedicals, Santa Ana, CA) in 3X and 5X volume to weight for iWAT and BAT samples, respectively, in cell lysis buffer (NP40 Cell Lysis Buffer (Life Technologies; CAT# FNN0021) supplemented with PMSF. Protein concentration of all homogenates were determined using a BCA assay and solubilized in Laemmli buffer (161-0747, BioRad). Specific protein loads, gel electrophoresis and antibody dilution information are summarized in **Table 1**. Membranes were imaged using Immobilon® ECL Ultra Western HRP Substrate (WBKLS0500, MilliporeSigma) and a BioRad ChemiDoc Imager. Target protein amounts were normalized to their respective total protein loads assessed *via* Ponceau staining. All images were quanitified using ImageLab software (BioRad).

**Table 1:** Western blotting electrophoresis and antibody details.

	Protein Loaded (µg)	Type of Gel	Membrane	Primary Antibody
SERCA1a	Soleus: 10 Adipose: 20	BioRad PreCast TGX 4-15% gradient gels	PVDF	MA3-912, ThermoFisher Scientific
SERCA2a	Soleus: 2.5 Adipose: 20	BioRad PreCast TGX 4-15% gradient gels	PVDF	MA3-919, ThermoFisher Scientific
RYR	Soleus: 10	Soleus: BioRad PreCast TGX 4-15% gradient gels	PVDF	MA3-925, ThermoFisher Scientific
SLN	Soleus: 25	BioRad PreCast TGX 4-Tricine	Nitrocellulose	ABT13, Sigma Aldrich
NNAT	Soleus: 10	BioRad PreCast TGX 4-15% gradient gels	PVDF	P127842, Cell Signaling
UCP1	Adipose: iWAT: 20 BAT: 5	BioRad PreCast TGX 4-15% gradient gels	PVDF	14670, Cell Signaling

SERCA1/2, sarco(endo)plasmic reticulum Ca^2+^ ATPase 1/2; RYR, ryanodine receptor; SLN, sarcolipin; NNAT, neuronatin; UCP1, uncoupling protein 1; PVDF, polyvinylidene difluoride.

Specific information regarding the protein load, electrophoresis, transfer, and primary antibody probes are presented for each protein target from muscle and adipose homogenate.

### Statistical analysis

All data are presented as mean± SEM. Statistical comparisons were made using either a Student’s t-test or a two-way ANOVA (main effects of genotype and either time or pCa, and their interaction). For ATP hydrolysis analyses, a test for a linear trend across increasing Ca2+ concentrations or pCa values of 6.10, 6.25, and 6.37 was performed for each genotype. For all analyses, outliers were detected and removed prior to analysis using the ROUT method (Q = 2%). All statistical analysis were done using Graphpad Prism 8 software with statistical significance set to a p < 0.05.

## Results

### Heterozygous neurogranin knockdown in the soleus muscles of female mice

**Figure 1:**
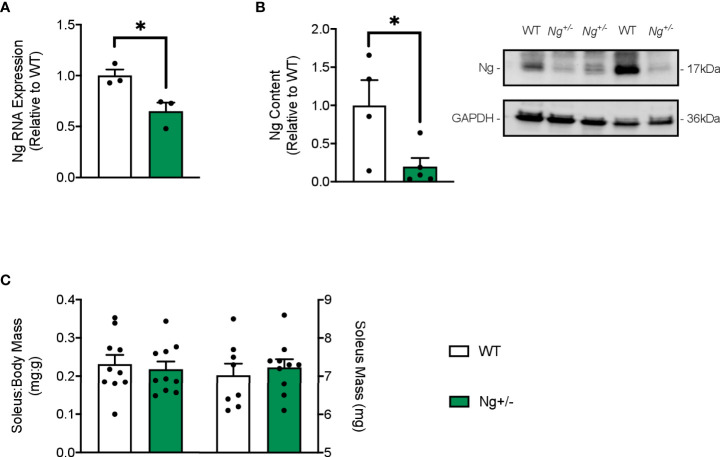
Neurogranin is reduced in the soleus muscles of *Ng^+/-^* mice but the soleus:body weight ratio is unchanged. Neurogranin expression in the soleus muscle of WT and *Ng^+/-^* mice was assessed *via* RT-PCR **(A)** and western blotting **(B)** and was found to be significantly reduced in *Ng^+/-^* mice compared to WT. The soleus:body weight ratios (mg:g) and soleus weights (mg) were unchanged between genotypes **(C)**. *p < 0.05 (n=3-4 per group (A,B); n=10 per group **(B)**.

Ng mRNA expression is significantly reduced (~35% reduction) in soleus muscles of Ng+/- mice compared to their WT littermates (**Figure 1A**). Corresponding well with this, we found a significant reduction in Ng protein in the soleus muscles from Ng+/- mice compared to WT (**Figure 1B**). No differences were observed between genotypes with regards to absolute or relative (to body mass) soleus weights (**Figure 1C**).

### Ng+/- mice have reduced physical activity but do not gain more weight on a HFD

**Figure 2:**
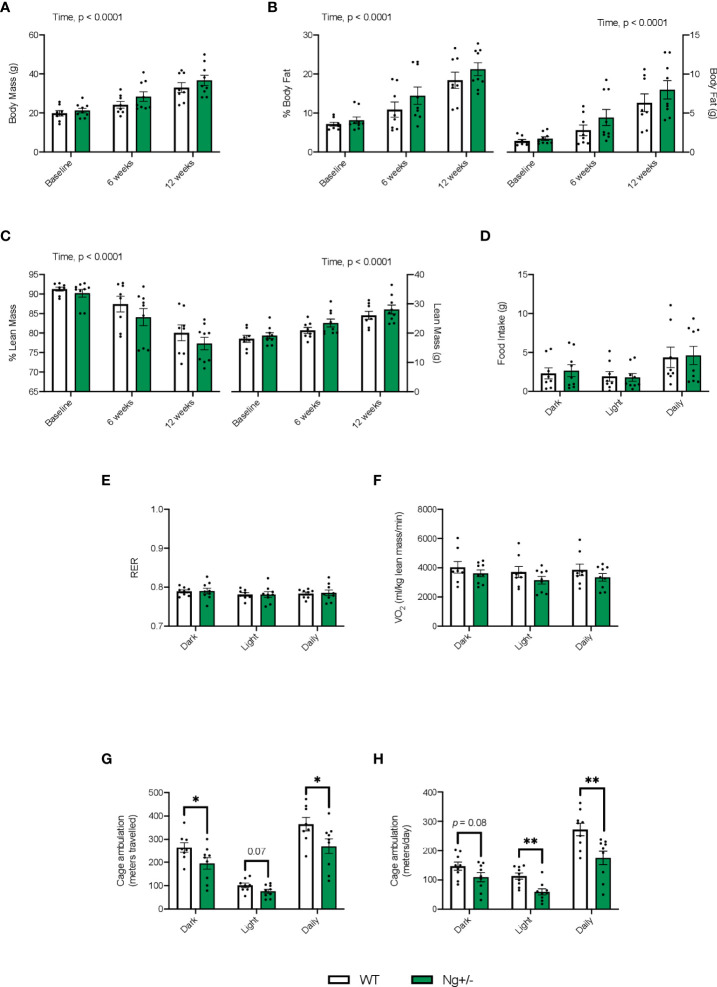
*Ng^+/-^* mice show significant reductions in cage ambulation but no changes in whole body metabolism. Both WT and *Ng^+/-^* mice show significant increases in body mass over the 12 week diet **(A)**. With increases in body mass we saw significant increases in % body fat **(B)** corresponding with significant reductions in % lean mass **(C)** across both genotypes over time. Body fat and lean mass (g) are also shown. No differences between food intake **(D)**, respiratory exchange ratio **(E)**, or volume of oxygen consumption (VO_2_, **F**) were detected across dark, light, and daily cycles nor between genotype. Cage ambulation measured using the DVC80 caging system, whereby cage activity was monitored 24/7, showed significant reductions in the dark and daily time points with the *Ng^+/-^* mice compared to WT with ambulation during the light cycles not reaching significance (*p* = 0.07, **G**). Similar results were found with the Promethion metabolic caging system, though no changes were seen in the dark cycle **(H)**. *p < 0.05, values above bars indicate *p* values (n=8-10 per group). **p < 0.01.

Both WT and Ng+/- mice had significant increases in body mass over the 12 week diet intervention with no differences observed between genotype (**Figure 2A**, main effect of time, p < 0.0001). Throughout the diet, both genotypes showed significant increases in % body fat (**Figure 2B**) and significant reductions in % lean mass (**Figure 2C**), with no effect of genotype detected (main effect of time, p < 0.0001). No differences were observed with food intake, RER, oxygen consumption (**Figures 2D–F**), or glucose and insulin tolerance (**Supplemental Figure 1**). Cage ambulation data showed significant reductions (p < 0.05) in meters travelled by the Ng+/- mice compared to WT in the dark cycle and daily period, with ambulation during the light cycles being reduced, but not reaching statistical significance (**Figure 2G**, p = 0.07). Similar findings were found using the Promethion metabolic cages, where Ng+/- mice were less active in their cages, and this was statistically significant in the light cycle and daily period (**Figure 2H**).

### Ng knockdown mice have reductions in SERCA’s apparent coupling ratio

**Figure 3:**
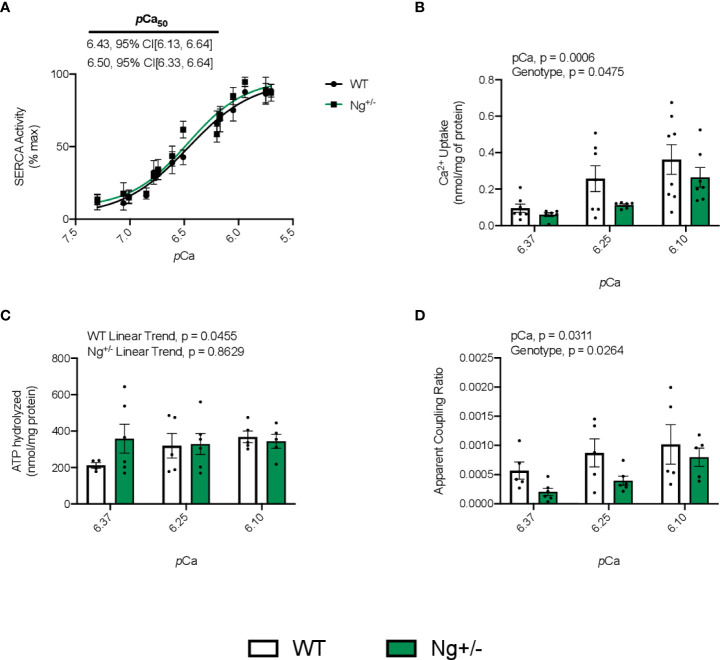
Soleus SERCA pump efficiency is reduced in *Ng^+/-^* mice compared to WT. Representative curves of SERCA ATPase activity with SERCA’s Ca^2+^ affinity measure, *p*Ca_50_, embedded with 95% confidence intervals **(A)**. Quantifying SERCA Ca^2+^ uptake over three different *p*Ca intervals (6.10, 6.25, 6.37) shows a significant main effect of *p*Ca and genotype **(B)**. Calculating the amount of ATP hydrolyzed across the same three *p*Ca intervals showed no main effects with a two-way ANOVA; however, testing for a linear trend within each genotype showed a significant linear trend of reduced ATPase activity with increased *p*Ca but this was not the case for *Ng^+/-^* mice **(C)**. Taking the ratio of Ca^2+^ uptake to ATP hydrolyzed at each *p*Ca concentration will give a measure of SERCA’s apparent coupling ratio. In doing so, we observed a significant main effect of both *p*Ca and genotype **(D)**. Values above bars indicate *p* values (n=5-8 per group).

SERCA’s apparent affinity for Ca2+ based on the SERCA activity – pCa curves was not different between genotypes (**Figure 3A**). Total Ca2+ uptake was calculated and normalized to mg of protein at 3 different time intervals throughout the uptake protocol: 1) 300-600s, 2) 600-900s, and 3) 900-1200s, which corresponded with an average pCa value of 6.10, 6.25, and 6.37 (**Figure 3B**). A significant main effect of pCa (p = 0.0006) was observed indicating that as free Ca2+ levels dropped, so too did the amount of Ca2+ uptake, highlighting the Ca2+ dependency of SERCA function. A main effect of genotype (p = 0.0475) was also detected indicating that soleus muscles from Ng+/- mice have less Ca2+ uptake compared to WT mice (**Figure 3B**). In calculating the amount of ATP hydrolyzed at the same 3 pCa values showed no significant main effects with a two-way ANOVA; however, linear trend analyses show a significant linear trend (p = 0.0455) of decreasing ATP hydrolysis with reductions in [Ca2+] for WT mice, which again highlights the Ca2+ dependency of SERCA function (**Figure 3C**). Interestingly, this was not observed in soleus muscles from Ng+/mice (p = 0.8629, **Figure 3C**). Finally, in taking the ratio of Ca2+ uptake to ATP hydrolyzed to calculate SERCA’s apparent coupling ratio at each pCa value, we see significant main effects of pCa (p = 0.0311) and genotype (p = 0.0264, **Figure 3D**), suggesting that SERCA is more efficient at higher free Ca2+ concentrations and that Ng+/- soleus have lower SERCA efficiency compared with WT.

### Monomeric phospholamban is reduced in soleus muscles of HFD-fed Ng+/- mice

**Figure 4:**
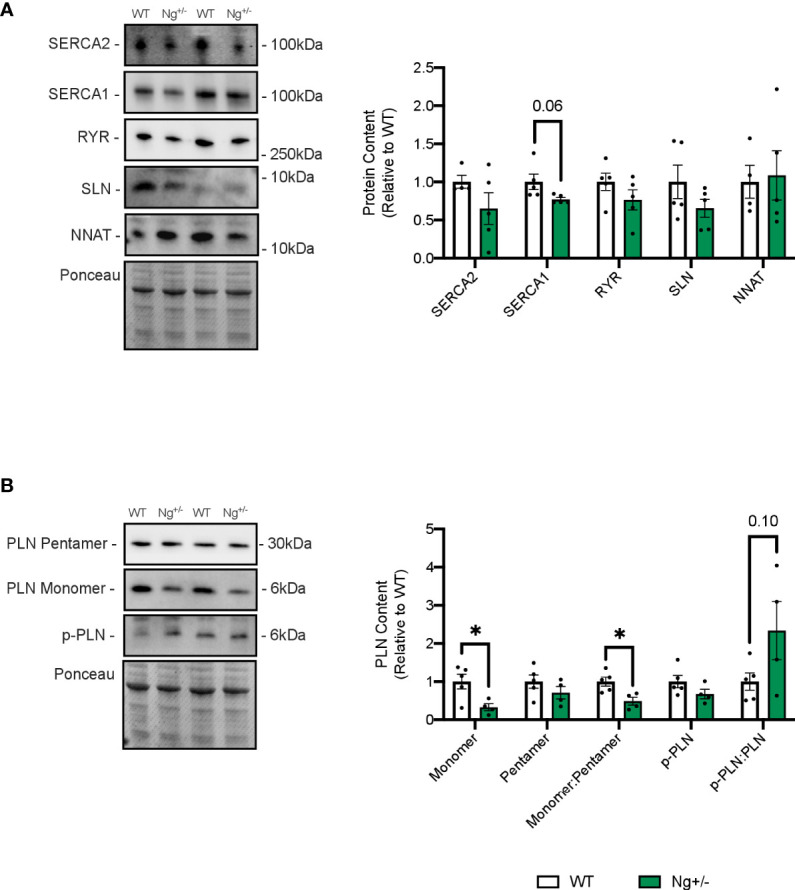
Phospholamban is reduced in the soleus muscles of *Ng^+/-^* mice. Representative Western blots and their corresponding analyses for SERCA2a, SERCA1a, RYR, SLN and NNAT in the soleus of WT and *Ng^+/-^* mice **(A)**. Only reductions in SERCA1a content were observed, though this did not reach statistical significance. Characterizing PLN and its activation status showed reductions in monomeric PLN resulting in reductions in the monomer:pentamer ratio and statistically insignificant increases in the p-PLN : PLN ratio **(B)**. Molecular weight markers are included in the representative blots. *p < 0.05, values above bars indicate *p* values (n=4-5 per group).

Western blotting was employed to investigate SERCA protein content, as well as its uncouplers SLN and NNAT (**Figure 4A**). RYR protein content was also investigated given its vital role in Ca2+ release, and leak, from the sarcoplasmic reticulum (SR) (40). No differences were seen between WT and Ng+/- mice for the SERCA2 or SERCA1 isoforms, RYR, nor SLN and NNAT (**Figure 4A**).

Next, the SERCA regulator phospholamban (PLN), which does not uncouple the SERCA pump was also investigated, and significant reductions in its active, monomeric form were observed (p < 0.05, **Figure 4B**). There were no changes in its inactive, pentameric form, resulting in a significant reduction in the monomer:pentamer ratio in Ng+/- soleus relative to WT (p < 0.05, **Figure 4B**). PLN can also be phosphorylated at two sites (Ser16, Thr17) to relieve inhibition on the SERCA pump (41). Phosphorylated PLN showed no differences between genotypes resulting in no statistical difference in the ratio of phosphorylated:active (**Figure 4B**).

### Heterozygous Ng deletion alters thermogenic protein content in white and brown adipose tissue

**Figure 5:**
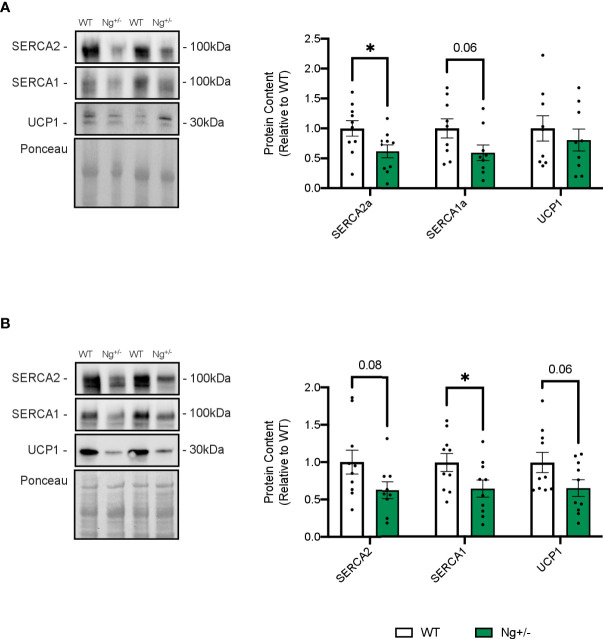
SERCA content is reduced in iWAT and BAT from *Ng^+/-^* mice. Representative Western blots and corresponding analyses of SERCA2a, SERCA1a, and UCP1 from iWAT **(A)** and BAT **(B)**. In both depots, reductions in both SERCA isoforms were observed with no changes in UCP1. *p < 0.05, values above bars indicate *p* values (n=9-10 per group).

Given that SERCA Ca2+ cycling has recently been shown to play a role in adipose tissue thermogenesis (42) in addition to UCP1, western blotting was employed to investigate SERCA and UCP1 protein content in iWAT (**Figure 5A**) and BAT (**Figure 5B**). SERCA2 and SERCA1 protein content are both reduced in Ng+/- iWAT (p < 0.05 and p = 0.06, respectively, **Figure 5A**) and BAT (p = 0.08 and p < 0.05, respectively, **Figure 5B**) compared to WT. No statistical differences were observed in UCP1 expression in iWAT (**Figure 5A**) or BAT in Ng+/- mice compared to WT (**Figure 5B**).

## Discussion

In this study, we investigated whether heterozygous Ng deletion would alter SERCA Ca2+ transport efficiency in female mice fed a HFD for 12 weeks. Based on our previous work showing that Ng negatively regulates calcineurin signaling, we hypothesized that SERCA efficiency would be lowered in soleus muscles from Ng+/- mice due to an increase in SLN expression. Our results show similar changes in body mass and composition between genotypes across the diet intervention, though the Ng+/- mice showed significant reductions in cage ambulation compared to WT mice across the 12 weeks. Despite this reduction in cage activity, WT and Ng+/- mice had similar levels of energy expenditure, food intake, glucose and insulin tolerance. Nonetheless, we found that SERCA’s apparent coupling ratio in the soleus was significantly lowered in Ng+/- mice compared to WT.

The reductions in cage ambulation observed in the Ng+/- mice compared to WT mice requires further investigation. While this is consistent with previous work in our lab with these mice (27), others have shown that Ng knockout mice exhibit traits of hyperactivity (43, 44). However, hyperactivity was only observed in an open field test, and not in a familiar environment such as their home cage (44). Nevertheless, our finding of reduced cage activity in heterozygous Ng knockdown mice is inconsistent with the previous literature (43, 44). It is worth noting that we received the Ng+/- mice *via* cryorecovery and have had difficulties in maintaining the breeding colony in terms of animal numbers and genotype. Specifically, breeding heterozygous mice with each other did not produce the expected number of homozygous Ng knockout mice with ~5% live births compared with the expected 25%. These limitations not only prevented us from using homozygous knockout mice but also limited our approach to solely HFD-fed groups. In any case, both heterozygous and homozygous genetic deletion of Ng has been shown to impair spatial memory of mice in a dose-dependent manner (23). However, with our cryorecovered mice, we did not find any deficits in spatial memory assessed through a novel object recognition test in the Ng+/- mice vs WT mice after a HFD (**Supplemental Figure 2**). The exact reasons for these discrepant findings remain unknown, however, we do note that previous studies have backcrossed the Ng knockout mice to a C57BL/6J background rather than the 129/Sv and C57BL/6J mixed background that was used for this present study (43, 44).

Even though the Ng+/- mice had reductions in physical activity, they did not gain more weight or fat mass compared to WT mice nor did they display any changes in glucose homeostasis. We suspect that part of this can be explained by changes in SERCA pump efficiency, as studies have shown that under HFD conditions, when SERCA pump efficiency is lowered (i.e., with SLN deletion) mice gain more fat mass and become more glucose intolerant (15, 19). Similarly, genetic deletion of another SERCA uncoupler, NNAT, increased diet induced-obesity and glucose intolerance (45). However, in contrast with our hypothesis, we did not find any changes in SLN or NNAT content. Originally, we hypothesized that Ng deletion, by virtue of enhanced calcineurin activation, would at the very least increase SLN content in the soleus to promote SERCA uncoupling. We have previously found that genetic reduction of Ng, both *in vitro* [C2C12 cells, (21)] and *in vivo* [male soleus, (27)], activates calcineurin signaling. Thus, it appears that the effects of Ng on SERCA efficiency and energy expenditure are independent of calcineurin.

We did observe reductions in SERCA1a protein content in the soleus of Ng+/- mice, which may be due to loss of Ng itself, fiber type changes, or reductions in cage activity, but we did not observe any differences in RYR content. Reductions in SERCA content without changes in RYR could alter how long Ca2+ remains in the cytosol considering SERCA density is a major determinant of the rate of Ca2+ uptake (46), resulting in an observation of reduced SERCA efficiency. Further, reductions in SERCA pump density with no changes in the uncouplers, SLN and NNAT, could alter the SERCA pump:uncoupler ratio in the SR leading to SERCA inefficiency, though this should be investigated further.

PLN is another SERCA regulatory protein that, unlike SLN and NNAT, does not uncouple the SERCA pump in skeletal muscle (47). Here, we found that monomeric PLN content was significantly reduced in soleus muscles from Ng+/- mice compared with WT. The monomeric form is known as the inhibitory form that binds to SERCA and reduces its affinity for Ca2+, whereas the pentameric form is thought to be an inactive storage form of PLN (48–50). Though purely speculative, a reduction in PLN content may also indirectly facilitate SERCA uncoupling by increasing the SERCA pump availability for SLN and NNAT, though we were limited in that we didn’t quantify SLN and NNAT binding to SERCA due to low sample volume. In addition to this, PLN’s inhibitory action on SERCA can be relieved through phosphorylation by protein kinase A and CaM kinase II (CaMKII) at its Ser16 and Thr17 sites, respectively (41, 51). Further, phosphorylation of PLN has been shown to increase passive Ca2+ leak from SR vesicles (52). Passive Ca2+ leak can also lower SERCA Ca2+ transport efficiency by reducing Ca2+ uptake into the SR and increasing Ca2+-dependent SERCA activity (16). Therefore, and though it was not statistically significant, the increase in PLN phosphorylation found in the Ng+/- soleus could also indirectly reduce SERCA’s Ca2+ transport efficiency; and would also be consistent with the role of Ng in sequestering CaM away from its cellular targets. That is, a reduction in Ng protein would increase CaM availability for CaMKII activation and subsequent PLN phosphorylation. Additionally, one study has shown that pentameric PLN is targeted for autophagic degradation (53) and CaM availability has been shown to be highly involved in promoting basal autophagic flux (54), potentially contributing to the reductions in PLN content observed in the present study.

In addition to skeletal muscle NST, adipose tissue, primarily BAT, can increase energy expenditure through UCP1 which acts to dissipate the mitochondrial proton gradient, uncoupling it from ATP synthesis and instead releasing the free energy as heat (55, 56). Importantly, WAT, traditionally thought of as a fat storage site, is not metabolically inert and can adopt a brown-like morphology under conditions of metabolic stress, such as diet-induced obesity (55). Since adipose tissue is another main site of NST, we also investigated iWAT and BAT SERCA and UCP1 content in WT and Ng+/- mice. We did observe reductions in both SERCA isoforms in each adipose depot, which is partly consistent with our findings in muscle, and we also saw reductions in UCP1 protein content in BAT. SERCA Ca2+ cycling has begun to be acknowledged as a thermogenic mechanism in adipose tissue in addition to UCP1 (42, 57, 58) and exercise has been shown to induce adipose browning and thermogenesis in iWAT (reviewed by (59)). While we are limited in the lack of adipose histology, we do acknowledge that it is possible that the reductions in physical activity in the Ng+/- mice is preventing increases in thermogenic proteins in iWAT. The effects of exercise on BAT activity and thermogenesis are still not fully elucidated (59), but the results presented in this study may suggest reduced contribution of adipose-Ca2+ cycling and UCP1-mediated thermogenesis in BAT in Ng+/- mice and this should be investigated further. Nevertheless, muscle-based SERCA Ca2+ cycling seems to counteract these reductions and prevent further weight gain.

While our study shows that heterozygous reduction of Ng alters SERCA efficiency in a manner independent of calcineurin signaling, we must acknowledge some key limitations. First, given the difficulties we faced while establishing the breeding colony, we were unable to perform experiments on homozygous Ng knockout mice. This limits our understanding of the role of Ng in skeletal muscle. Future studies with muscle-specific deletion of Ng would provide additional mechanistic insight. Secondly, due to these limitations we were unable to include a control diet group and, at best, were only able to include some longitudinal data (i.e., body composition at baseline vs body composition post-high-fat diet). Thirdly, we chose to examine only the soleus based on the relative expression of not only Ng (21), but also the two uncouplers SLN and NNAT, both of which appear to be greater in the soleus vs the fast-twitch extensor digitorum longus (EDL) (31, 60). Thus, our study is limited in the fact that we did not assess the effects of Ng knockdown on other types of muscles. Furthermore, while we have previously shown that soleus muscles from Ng+/- mice are more fatigue resistant due to an increase in calcineurin activation and promotion of the oxidative fibre type when compared with WT controls, we did not assess skeletal muscle contractility in this study. This is important as a previous study by Funai etal. (61) showed that lowered SERCA efficiency, caused by altered SR phospholipid composition from a HFD, prevented diet-induced diabetes but led to muscle weakness – representing an important trade-off when targeting SERCA efficiency in muscle for metabolic disorders such as obesity. Lastly, our study was conducted in standard room temperature conditions, and the impact of housing temperature on rodent muscle metabolism is becoming increasingly known as of late. It is possible that the mild cold stress induced by housing mice at room temperature could have confounded or masked any effect of Ng deletion on SERCA efficiency, energy expenditure, changes in body mass and glucose homeostasis.

Nonetheless, and in conclusion, this study found that despite heterozygous Ng+/- mice moving less in their cages, there were no differences in body mass, composition, energy expenditure, glucose or insulin tolerance compared with WT mice after being fed a high fat diet. Furthermore, we found that heterozygous deletion of Ng in the soleus promoted SERCA uncoupling in a manner independent of any changes in SLN or NNAT, but one associated with a reduction in SERCA1 and PLN.

## Data availability statement

The raw data supporting the conclusions of this article will be made available by the authors, without undue reservation.

## Ethics statement

The animal study was reviewed and approved by Brock University Animal Care Committee.

## Author contributions

JB: conceptualization, investigation, methodology, formal analysis, data curation, visualization, writing – original draft, writing – review & editing. JR and KG: investigation, methodology, data curation, writing – review & editing. EC, MG, and RB: investigation, data curation, writing – review & editing. RM: funding acquisition, resources, supervision, writing – review & editing. VF: conceptualization, formal analysis, visualization, project administration, funding acquisition, supervision, writing – original draft, writing – review & editing. All authors contributed to the article and approved the submitted version.

## Funding

JB was supported by a Canadian Institutes of Health Research (CIHR) CGS-M award. JR was supported by a Natural Sciences and Engineering Research Council of Canada (NSERC) undergraduate student research award. This work was supported by an NSERC Discovery Grant to VF. VF is supported by a Canada Research Chair (Tier 2) Award.

## Acknowledgments

We thank the animal care technicians at Brock University.

## Conflict of interest

The authors declare that the research was conducted in the absence of any commercial or financial relationships that could be construed as a potential conflict of interest.

## Publisher’s note

All claims expressed in this article are solely those of the authors and do not necessarily represent those of their affiliated organizations, or those of the publisher, the editors and the reviewers. Any product that may be evaluated in this article, or claim that may be made by its manufacturer, is not guaranteed or endorsed by the publisher.

## References

[1] Chin ER Olson EN Richardson JA Yang Q Humphries C Shelton JM. A calcineurin-dependent transcriptional pathway controls skeletal muscle fiber type. Genes Dev (1998) 12:2499–509. doi: 10.1101/gad.12.16.2499 PMC317085 9716403

[2] Mccullagh KJ Calabria E Pallafacchina G Ciciliot S Serrano AL Argentini C. NFAT is a nerve activity sensor in skeletal muscle and controls activity-dependent myosin switching. Proc Natl Acad Sci USA(2004) 101:10590–5. doi: 10.1073/pnas.0308035101 PMC489979 15247427

[3] Long YC Glund S Garcia-Roves PM Zierath JR. Calcineurin regulates skeletal muscle metabolism *via* coordinated changes in gene expression. J Biol Chem (2007) 282:1607–14. doi: 10.1074/jbc.M609208200 17107952

[4] Chen HH Chen WP Yan WL Huang YC Chang SW Fu WM. NRIP is newly identified as a z-disc protein, activating calmodulin signaling for skeletal muscle contraction and regeneration. J Cell Sci (2015) 128:4196–209. doi: 10.1242/dev.133009 26430214

[5] Moradi F Copeland EN Baranowski RW Scholey AE Stuart JA Fajardo VA. Calmodulin-binding proteins in muscle: A minireview on nuclear receptor interacting protein, neurogranin, and growth-associated protein 43. Int J Mol Sci (2020) 21:1–12. doi: 10.3390/ijms21031016 PMC7038096 32033037

[6] Dunlap TB Cook EC Rumi-Masante J Arvin HG Lester TE Creamer TP. The distal helix in the regulatory domain of calcineurin is important for domain stability and enzyme function. Biochemistry (2013) 52:8643–51. doi: 10.1021/bi400483a 24191726

[7] Whitley KC Hamstra SI Baranowski RW Watson CJF Macpherson REK Macneil AJ. GSK3 inhibition with low dose lithium supplementation augments murine muscle fatigue resistance and specific force production. Physiol Rep (2020) 8:e14517. doi: 10.14814/phy2.14517 32729236 PMC7390913

[8] Chakkalakal JV Stocksley MA Harrison MA Angus LM Deschenes-Furry J St-Pierre S. Expression of utrophin a mRNA correlates with the oxidative capacity of skeletal muscle fiber types and is regulated by calcineurin/NFAT signaling. Proc Natl Acad Sci USA (2003) 100:7791–6. doi: 10.1073/pnas.0932671100 PMC164666 12808150

[9] Chakkalakal JV Harrison MA Carbonetto S Chin E Michel RN Jasmin BJ. Stimulation of calcineurin signaling attenuates the dystrophic pathology in mdx mice. Hum Mol Genet (2004) 13:379–88. doi: 10.1093/hmg/ddh037 14681302

[10] Rotter D Peiris H Grinsfelder DB Martin AM Burchfield J Parra V. Regulator of calcineurin 1 helps coordinate whole-body metabolism and thermogenesis. EMBO Rep (2018) 19:1–19. doi: 10.15252/embr.201744706 PMC6280800 30389725

[11] Nowack J Giroud S Arnold W Ruf T. Muscle non-shivering thermogenesis and its role in the evolution of endothermy. Front Physiol (2017) 8:889. doi: 10.3389/fphys.2017.00889 29170642 PMC5684175

[12] Li H Wang C Li L Li L. Skeletal muscle non-shivering thermogenesis as an attractive strategy to combat obesity. Life Sci (2021) 269:119024. doi: 10.1016/j.lfs.2021.119024 33450257

[13] Toyoshima C Nakasako M Nomura H Ogawa H. Crystal structure of the calcium pump of sarcoplasmic reticulum at 2.6 a resolution. Nature (2000) 405:647–55. doi: 10.1038/35015017 10864315

[14] Toyoshima C. Ion pumping by calcium ATPase of sarcoplasmic reticulum. Adv Exp Med Biol (2007) 592:295–303. doi: 10.1007/978-4-431-38453-3_25 17278374

[15] Bombardier E Smith IC Vigna C Fajardo VA Tupling AR. Ablation of sarcolipin decreases the energy requirements for Ca2+ transport by sarco(endo)plasmic reticulum Ca2+-ATPases in resting skeletal muscle. FEBS Lett (2013) 587:1687–92. doi: 10.1016/j.febslet.2013.04.019 23628781

[16] Gamu D Juracic ES Hall KJ Tupling AR. The sarcoplasmic reticulum and SERCA: a nexus for muscular adaptive thermogenesis. Appl Physiol Nutr Metab (2020) 45:1–10. doi: 10.1139/apnm-2019-0067 31116956

[17] Mengeste AM Katare P Dalmao Fernandez A Lund J Bakke HG Baker D. Knockdown of sarcolipin (SLN) impairs substrate utilization in human skeletal muscle cells. Mol Biol Rep (2022) 49(7):6005–17. doi: 10.1007/s11033-022-07387-0 PMC9270280 35364719

[18] Mall S Broadbridge R Harrison SL Gore MG Lee AG East JM. The presence of sarcolipin results in increased heat production by Ca(2+)-ATPase. J Biol Chem (2006) 281:36597–602. doi: 10.1074/jbc.M606869200 17018526

[19] Bal NC Maurya SK Sopariwala DH Sahoo SK Gupta SC Shaikh SA. Sarcolipin is a newly identified regulator of muscle-based thermogenesis in mammals. Nat Med (2012) 18:1575–9. doi: 10.1038/nm.2897 PMC3676351 22961106

[20] Braun JL Teng ACT Geromella MS Ryan CR Fenech RK Macpherson REK. Neuronatin promotes SERCA uncoupling and its expression is altered in skeletal muscles of high-fat diet-fed mice. FEBS Lett (2021) 595:2756–67. doi: 10.1002/1873-3468.14213 34693525

[21] Fajardo VA Watson CJF Bott KN Moradi F Maddalena LA Bellissimo CA. Neurogranin is expressed in mammalian skeletal muscle and inhibits calcineurin signaling and myoblast fusion. Am J Physiol Cell Physiol (2019) 317:C1025–33. doi: 10.1152/ajpcell.00345.2018 31433693

[22] Martzen MR Slemmon JR. The dendritic peptide neurogranin can regulate a calmodulin-dependent target. J Neurochem (1995) 64:92–100. doi: 10.1046/j.1471-4159.1995.64010092.x 7528268

[23] Pak JH Huang FL Li J Balschun D Reymann KG Chiang C. Involvement of neurogranin in the modulation of calcium/calmodulin-dependent protein kinase II, synaptic plasticity, and spatial learning: a study with knockout mice. Proc Natl Acad Sci USA (2000) 97:11232–7. doi: 10.1073/pnas.210184697 PMC17183 11016969

[24] Slemmon JR Feng B Erhardt JA. Small proteins that modulate calmodulin-dependent signal transduction: effects of PEP-19, neuromodulin, and neurogranin on enzyme activation and cellular homeostasis. Mol Neurobiol (2000) 22:99–113. doi: 10.1385/MN:22:1-3:099 11414283

[25] Gaertner TR Putkey JA Waxham MN. RC3/Neurogranin and Ca2+/calmodulin-dependent protein kinase II produce opposing effects on the affinity of calmodulin for calcium. J Biol Chem (2004) 279:39374–82. doi: 10.1074/jbc.M405352200 15262982

[26] Li L Lai M Cole S Le Novère N Edelstein SJ. Neurogranin stimulates Ca2+/calmodulin-dependent kinase II by suppressing calcineurin activity at specific calcium spike frequencies. PloS Comput Biol (2020) 16:e1006991. doi: 10.1371/journal.pcbi.1006991 32049957 PMC7041932

[27] Baranowski RW Braun JL Vandenboom R Fajardo VA. Neurogranin inhibits calcineurin in murine soleus muscle: Effects of heterozygous knockdown on muscle adaptations to tenotomy and fatigue resistance. Biochem Biophys Res Commun (2022) 623:89–95. doi: 10.1016/j.bbrc.2022.07.062 35878428

[28] Hayward GC Caceres D Copeland EN Baranowski BJ Mohammad A Whitley KC. Characterization of alzheimer's disease-like neuropathology in duchenne's muscular dystrophy using the DBA/2J mdx mouse model. FEBS Open Bio (2022) 12:154–62. doi: 10.1002/2211-5463.13317 PMC8727939 34668666

[29] Schiaffino S Reggiani C. Fiber types in mammalian skeletal muscles. Physiol Rev (2011) 91:1447–531. doi: 10.1152/physrev.00031.2010 22013216

[30] Vangheluwe P Schuermans M Zador E Waelkens E Raeymaekers L Wuytack F. Sarcolipin and phospholamban mRNA and protein expression in cardiac and skeletal muscle of different species. Biochem J (2005) 389:151–9. doi: 10.1042/BJ20050068 PMC1184547 15801907

[31] Braun JL Geromella MS Hamstra SI Fajardo VA. Neuronatin regulates whole-body metabolism: is thermogenesis involved? FASEB Bioadv (2020) 2:579–86. doi: 10.1096/fba.2020-00052 PMC7566048 33089074

[32] Wan Z Ritchie I Beaudoin MS Castellani L Chan CB Wright DC. IL-6 indirectly modulates the induction of glyceroneogenic enzymes in adipose tissue during exercise. PloS One (2012) 7:e41719. doi: 10.1371/journal.pone.0041719 22844518 PMC3402468

[33] Macpherson RE Baumeister P Peppler WT Wright DC Little JP. Reduced cortical BACE1 content with one bout of exercise is accompanied by declines in AMPK, akt, and MAPK signaling in obese, glucose-intolerant mice. J Appl Physiol (1985) (2015) 119:1097–104. doi: 10.1152/japplphysiol.00299.2015 PMC4816412 26404616

[34] Nolan T Hands RE Bustin SA. Quantification of mRNA using real-time RT-PCR. Nat Protoc (2006) 1:1559–82. doi: 10.1038/nprot.2006.236 17406449

[35] Tupling R Green H. Silver ions induce Ca2+ release from the SR *in vitro* by acting on the Ca2+ release channel and the Ca2+ pump. J Appl Physiol (1985) (2002) 92:1603–10. doi: 10.1152/japplphysiol.00756.2001 11896027

[36] Fajardo VA Bombardier E Mcmillan E Tran K Wadsworth BJ Gamu D. Phospholamban overexpression in mice causes a centronuclear myopathy-like phenotype. Dis Model Mech (2015) 8:999–1009. doi: 10.1242/dmm.020859 26035394 PMC4527296

[37] Fajardo VA Smith IC Bombardier E Chambers PJ Quadrilatero J Tupling AR. Diaphragm assessment in mice overexpressing phospholamban in slow-twitch type I muscle fibers. Brain Behav (2016) 6:e00470. doi: 10.1002/brb3.470 27134770 PMC4842933

[38] Duhamel TA Green HJ Stewart RD Foley KP Smith IC Ouyang J. Muscle metabolic, SR Ca(2+) -cycling responses to prolonged cycling, with and without glucose supplementation. J Appl Physiol (1985) (2007) 103:1986–98. doi: 10.1152/japplphysiol.01440.2006 17916667

[39] Braun JL Hamstra SI Messner HN Fajardo VA. SERCA2a tyrosine nitration coincides with impairments in maximal SERCA activity in left ventricles from tafazzin-deficient mice. Physiol Rep (2019) 7:e14215. doi: 10.14814/phy2.14215 31444868 PMC6708055

[40] Lanner JT Georgiou DK Joshi AD Hamilton SL. Ryanodine receptors: structure, expression, molecular details, and function in calcium release. Cold Spring Harb Perspect Biol (2010) 2:a003996. doi: 10.1101/cshperspect.a003996 20961976 PMC2964179

[41] Koss KL Kranias EG. Phospholamban: a prominent regulator of myocardial contractility. Circ Res (1996) 79:1059–63. doi: 10.1161/01.RES.79.6.1059 8943944

[42] Ikeda K Kang Q Yoneshiro T Camporez JP Maki H Homma M. UCP1-independent signaling involving SERCA2b-mediated calcium cycling regulates beige fat thermogenesis and systemic glucose homeostasis. Nat Med (2017) 23:1454–65. doi: 10.1038/nm.4429 PMC5727902 29131158

[43] Huang FL Huang KP. Methylphenidate improves the behavioral and cognitive deficits of neurogranin knockout mice. Genes Brain Behav (2012) 11:794–805. doi: 10.1111/j.1601-183X.2012.00825.x 22809330 PMC3467336

[44] Nakajima R Hattori S Funasaka T Huang FL Miyakawa T. Decreased nesting behavior, selective increases in locomotor activity in a novel environment, and paradoxically increased open arm exploration in neurogranin knockout mice. Neuropsychopharmacol Rep (2021) 41:111–6. doi: 10.1002/npr2.12150 PMC8182962 33270377

[45] Millership SJ Tunster SJ Van De Pette M Choudhury AI Irvine EE Christian M. Neuronatin deletion causes postnatal growth restriction and adult obesity in 129S2/Sv mice. Mol Metab (2018) 18:97–106. doi: 10.1016/j.molmet.2018.09.001 30279096 PMC6308027

[46] Tupling AR. The sarcoplasmic reticulum in muscle fatigue and disease: role of the sarco(endo)plasmic reticulum Ca2+-ATPase. Can J Appl Physiol (2004) 29:308–29. doi: 10.1139/h04-021 15199229

[47] Gamu D Juracic ES Fajardo VA Rietze BA Tran K Bombardier E. Phospholamban deficiency does not alter skeletal muscle SERCA pumping efficiency or predispose mice to diet-induced obesity. Am J Physiol Endocrinol Metab (2019) 316:E432–e442. doi: 10.1152/ajpendo.00288.2018 30601702

[48] Kimura Y Kurzydlowski K Tada M Maclennan DH. Phospholamban inhibitory function is activated by depolymerization. J Biol Chem (1997) 272:15061–4. doi: 10.1074/jbc.272.24.15061 9182523

[49] Maclennan DH Kranias EG. Phospholamban: a crucial regulator of cardiac contractility. Nat Rev Mol Cell Biol (2003) 4:566–77. doi: 10.1038/nrm1151 12838339

[50] Gorski PA Ceholski DK Young HS. Structure-function relationship of the SERCA pump and its regulation by phospholamban and sarcolipin. Adv Exp Med Biol (2017) 981:77–119. doi: 10.1007/978-3-319-55858-5_5 29594859

[51] Gustavsson M Verardi R Mullen DG Mote KR Traaseth NJ Gopinath T. Allosteric regulation of SERCA by phosphorylation-mediated conformational shift of phospholamban. Proc Natl Acad Sci USA (2013) 110:17338–43. doi: 10.1073/pnas.1303006110 PMC3808617 24101520

[52] Aschar-Sobbi R Emmett TL Kargacin GJ Kargacin ME. Phospholamban phosphorylation increases the passive calcium leak from cardiac sarcoplasmic reticulum. Pflugers Arch (2012) 464:295–305. doi: 10.s1007/00424-012-1124-9 22772476

[53] Teng AC Miyake T Yokoe S Zhang L Rezende LM Jr. Sharma P. Metformin increases degradation of phospholamban *via* autophagy in cardiomyocytes. Proc Natl Acad Sci USA (2015) 112:7165–70. doi: 10.1073/pnas.1508815112 PMC4466728 26040000

[54] Giles J Lopez V Mcconnaha E Hayden M Kragenbring C Carli D. Regulation of basal autophagy by calmodulin availability. FEBS J (2022). doi: 10.1111/febs.16432 35285161

[55] Rosen ED Spiegelman BM. What we talk about when we talk about fat. Cell (2014) 156:20–44. doi: 10.1016/j.cell.2013.12.012 24439368 PMC3934003

[56] Chouchani ET Kazak L Spiegelman BM. New advances in adaptive thermogenesis: UCP1 and beyond. Cell Metab (2019) 29:27–37. doi: 10.1016/j.cmet.2018.11.002 30503034

[57] Mottillo EP Ramseyer VD Granneman JG. SERCA2b cycles its way to UCP1-independent thermogenesis in beige fat. Cell Metab (2018) 27:7–9. doi: 10.1016/j.cmet.2017.12.015 29320712

[58] Guarnieri AR Benson TW Tranter M. Calcium cycling as a mediator of thermogenic metabolism in adipose tissue. Mol Pharmacol (2022) 102(1):51–9. doi: 10.1124/molpharm.121.000465 PMC9341262 35504660

[59] Vidal P Stanford KI. Exercise-induced adaptations to adipose tissue thermogenesis. Front Endocrinol (Lausanne) (2020) 11:270. doi: 10.3389/fendo.2020.00270 32411099 PMC7201000

[60] Tupling AR Bombardier E Gupta SC Hussain D Vigna C Bloemberg D. Enhanced Ca2+ transport and muscle relaxation in skeletal muscle from sarcolipin-null mice. Am J Physiol Cell Physiol (2011) 301:C841–849. doi: 10.1152/ajpcell.00409.2010 PMC3654932 21697544

[61] Funai K Song H Yin L Lodhi IJ Wei X Yoshino J. Muscle lipogenesis balances insulin sensitivity and strength through calcium signaling. J Clin Invest (2013) 123:1229–40. doi: 10.1172/JCI65726 PMC3582136 23376793

