# Corringendum: The effects of neurogranin knockdown on SERCA pump efficiency in soleus muscles of female mice fed a high fat diet

**DOI:** 10.3389/fendo.2022.1037434

**Published:** 2022-09-21

**Authors:** Jessica L. Braun, Jisook Ryoo, Kyle Goodwin, Emily N. Copeland, Mia S. Geromella, Ryan W. Baranowski, Rebecca E. K. MacPherson, Val A. Fajardo

**Affiliations:** ^1^ Department of Kinesiology, Brock University, St. Catharines, ON, Canada; ^2^ Centre for Bone and Muscle Health, Brock University, St. Catharines, ON, Canada; ^3^ Centre for Neuroscience, Brock University, St. Catharines, ON, Canada; ^4^ Department of Health Sciences, Brock University, St. Catharines, ON, Canada

**Keywords:** calcineurin, calmodulin, sarcolipin, neuronatin, phospholamban, obesity

## Text correction

In the published article, there was an error in the text. The source of the neurogranin mouse colony was incorrect. A correction has been made to the Methods, *animals*, section. This sentence previously stated:

“[A breeding colony of heterozygous Ng knockout mice (Ng+/-) and wild-type (WT) mice on a 129/Sv and C57BL/6J mixed background was established at Brock University using cryorecovered breeding pairs from the Mutant Mouse Resource and Research Centre (mmRRC, stock#043288-MU).]”

The corrected sentence appears below:

“[A breeding colony of heterozygous Ng knockout mice (Ng+/-) and wild-type (WT) mice on a 129/Sv and C57BL/6J mixed background was established at Brock University using cryorecovered breeding pairs from the Jackson Laboratories (stock#008233).]”

The authors apologize for this error and state that this does not change the scientific conclusions of the article in any way. The original article has been updated.

## Publisher’s note

All claims expressed in this article are solely those of the authors and do not necessarily represent those of their affiliated organizations, or those of the publisher, the editors and the reviewers. Any product that may be evaluated in this article, or claim that may be made by its manufacturer, is not guaranteed or endorsed by the publisher.

