# Supplementary material for: The effects of neurogranin knockdown on SERCA pump efficiency in soleus muscles of female mice fed a high fat diet
Source: Front Endocrinol (Lausanne). 2022 Aug 22;13:957182. doi: 10.3389/fendo.2022.957182 (PMC9441848; doi:10.3389/fendo.2022.957182)
Supplement: Supplementary file 1 [file DataSheet_1.docx]

**The effects of neurogranin knockdown on SERCA pump efficiency in female mice fed a high fat diet**

Supplemental Information

Jessica L. Braun^a,b,c^, Jisook Ryoo^a,b^, Kyle Goodwin^a,b^, Emily C. Copeland^a,b,c^, Mia S. Geromella^a,b^, Ryan W. Baranowski^a,b^, Rebecca E.K. MacPherson^c,d^, Val A. Fajardo^a,b,c*^

^a^Department of Kinesiology, Brock University, St. Catharines, ON L2S 3A1, Canada; [jb15gq@brocku.ca](mailto:jb15gq@brocku.ca) (J.L.B.); [jr12ft@brocku.ca](mailto:jr12ft@brocku.ca) (J.R.); [kg17jm@brocku.ca](mailto:kg17jm@brocku.ca) (K.G.); [rb15xz@brocku.ca](mailto:rb15xz@brocku.ca) (R.W.B.); [mg14dd@brocku.ca](mailto:mg14dd@brocku.ca) (M.S.G.)

^b^Centre for Bone and Muscle Health, Brock University, St. Catharines, ON L2S 3A1, Canada

^c^Centre for Neuroscience, Brock University, St. Catharines, ON L2S 3A1, Canada

^d^Department of Health Sciences, Brock University, St. Catharines, ON L2S 3A1, Canada; [rmacpherson@brocku.ca](mailto:rmacpherson@brocku.ca) (R.E.K.M.)

*Corresponding author: [vfajardo@brocku.ca](mailto:vfajardo@brocku.ca) (V.A.F.)

^^

**Supplemental Figure 1. Glucose and insulin tolerance tests in WT and *Ng^+/-^* mice (n = 10 per group).** Blood glucose was plotted over time for both WT and *Ng^+/-^* mice following a glucose injection (A). AUC analyses showed no differences between the genotypes (B). The same was plotted after an insulin injection (C) where again no differences in AUC was observed (D).

**Supplemental Figure 2. Percent of time investigating a novel object (n = 10 per group).** The time spent investigating a novel object, as measured by the novel object recognition test, and presented as a % of the total time investigating (time with novel object/(time with novel object + time with familiar object). No differences were observed between WT and *Ng^+/-^* mice.


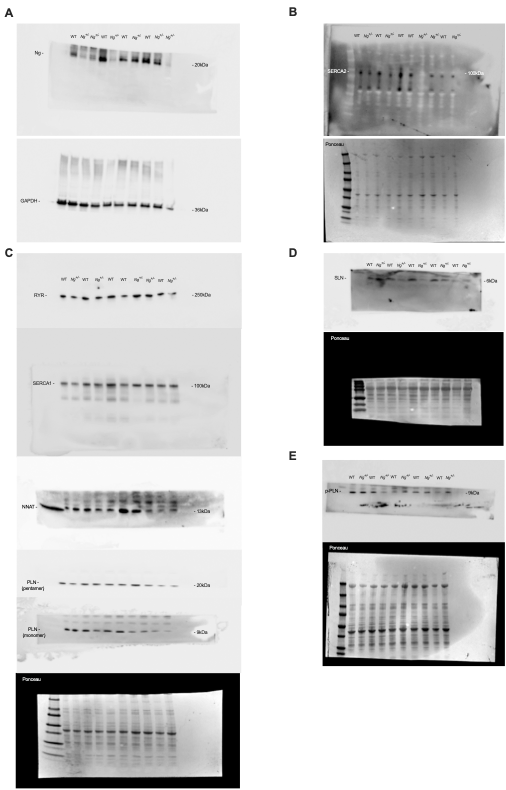


**Supplemental Figure 3. Full blot images from the soleus muscle.** Full, uncropped images of Western blots performed on the soleus muscle. Ng content was normalized relative to GAPDH (A) whereas SERCA2 (B), RYR, SERCA1, NNAT, PLN, and SLN (C, D, E) were all made relative to their respective Ponceau stains for total protein.


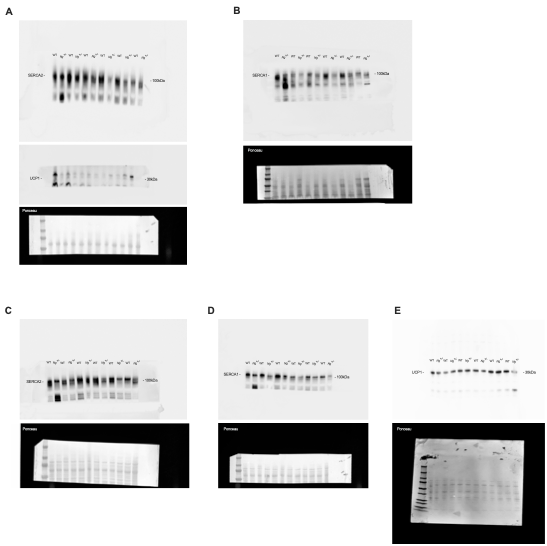


**Supplemental Figure 4. Full blot images from iWAT and BAT.** Full, uncropped images of Western blots performed on both iWAT and BAT. iWAT blots for SERCA2, UCP1 (A) and SERCA1 (B) are shown with their respective ponceaus. The same are shown for BAT in C, D, and E.
